# Supplementary material for: l-Asparaginase Type II from Fusarium proliferatum: Heterologous Expression and In Silico Analysis
Source: Pharmaceutics. 2023 Sep 20;15(9):2352. doi: 10.3390/pharmaceutics15092352 (PMC10534586; doi:10.3390/pharmaceutics15092352)
Supplement: Supplementary file 1 [file pharmaceutics-15-02352-s001.zip › Supplementary material.pdf]

Supplementary material

Supplementary Figure S1

```
B2022031892C7BAECDB1C.. Query     1  ------------------------NLPNITIYATGGTIAGSASSADQTTGYQSAALSVQS    36
I1SBD9 I1SBD9_PECAS     48.0%     1  ---MFNALFVVVFCFSSLANAAENLPNIVILATGGTIAGSAAANTQTTGYKAGALGVET    57
P06608 ASPG_DICCH       46.5%     1  MERWFKSLFVLV-LFFVFTASAADKLPNIVILATGGTIAGSAATGTQTTGYKAGALGVDT    59
Q0PC96 Q0PC96_CAMJE     43.3%     1  ---------------------MKKAKSRIAILGTGGTIAGFIDSTIATTGYAAGAIDIDV    39
P00805 ASPG2_ECOLI      39.8%     1  MEFFKKTALAAL---VMGFSGAALALPNITILATGGTIAGGGDSAT-KSNYTVGKVGVEN    56
P10182 ASPQ_PSES7       38.4%     1  -----------------KEVENQQKLANVVILATGGTIAGAGASAANSATYQAAKVGVDK    43
P10172 ASPQ_ACIGL       37.7%     1  -------------------------KNNVVIVATGGTIAGAGASSTNSATYSAAKVPVDA    35
P50286 ASPG_WOLSU       36.6%     1  -----------------------MAKPQVTILATGGTIAGSGESSV-KSSYSAGAVTVDK    36
                                                                .:.* .*******   :   .: *  . : ::
                        Similarity

B2022031892C7BAECDB1C..          37  LIDAVPQLCNVANVRGVQFANTDSIDMSSDMLQALVEQIQADLDNPSTQGAVVTHGTDTL    96
I1SBD9 I1SBD9_PECAS             58  LIQAVPELKTLANIKGEQVASIGSENMTSDVLLTLSKRVNELLARSDVDGVVITHGTDTL   117
P06608 ASPG_DICCH               60  LINAVPEVKKLANVKGEQFSNMASENMTGDVVLKLSQRVNELLARDDVDGVVITHGTDTV   119
Q0PC96 Q0PC96_CAMJE             40  LIKAVPQIRDLADISWEQIANIDSSNMCDEIWLRLAKKIAKLFA-EGIDGVVITHGTDTM    98
P00805 ASPG2_ECOLI              57  LVNAVPQLKDIANVKGEQVVNIGSQDMNDNVWLTLAKKINTDC--DKTDGFVITHGTDTM   114
P10182 ASPQ_PSES7               44  LIAGVPELADLANVRGEQVMQIASESITNDDLLKLGKRVAELADSNDVDGIVITHGTDTL   103
P10172 ASPQ_ACIGL               36  LIKAVPQVNDLANITGIQALQVASESITDKELLSLARQVNDLVKKPSVNGVVITHGTDTM    95
P50286 ASPG_WOLSU               37  LLAAVPAINDLATIKGEQISSIGSQEMTGKVWLKLAKRVNELLAQKETEAVIITHGTDTM    96
                                     *: .** :  :* :   *  .  * .: ..    * .::         :. ::******:

B2022031892C7BAECDB1C..          97  DESAFFLDLTIQSDKPVVVTGSMRPATAISADGPMNLLSSVTLAANENARGRGVMIAIND   156
I1SBD9 I1SBD9_PECAS            118  DESPYFLNLTVKSDKPVVFVAAMRPATAISADGPMNLYGAVKVAADKNSRGRGVLVVLND   177
P06608 ASPG_DICCH              120  EESAYFLHLTVKSDKPVVFVAAMRPATAISADGPMNLLEAVRVAGDKQSRGRGVMVVLND   179
Q0PC96 Q0PC96_CAMJE             99  EETAYFLNLTIKSDKPVVLVGAMRPSTAISADGPKNLYNAVALVVNKEAKNKGVMVAIND   158
P00805 ASPG2_ECOLI             115  EETAYFLDLTVKCDKPVVMVGAMRPSTSMSADGPFNLYNAVVTAADKASANRGVLVVMND   174
P10182 ASPQ_PSES7              104  EETAYFLDLTLNTDKPIVVVGSMRPGTAMSADGMLNLYNAVAVASNKDSRGKGVLVTMND   163
P10172 ASPQ_ACIGL               96  EETAFFLNLVVHTDKPIVLVGSMRPSTALSADGPLNLYSAVALASSNEAKNKGVMVLMND   155
P50286 ASPG_WOLSU               97  EETAFFLNLTVKSQKPVVLVGAMRSGSSMSADGPMNLYNAVNVAINKASTNKGVVIVMND   156
                                     :*: :**.*.:: :**:*...:** .:::****  **  :*  . .: : .:**:: :**

B2022031892C7BAECDB1C..         157  RIGSARFTTKVNANHLDAFQAPDSGLLGTFVNIQPVFFYPPSRPLGHHHFKLPPRPPSAA   216
I1SBD9 I1SBD9_PECAS            178  RIGSARFISKTNASTLDTFKAPEEGYLGVIIGDKIYYQTRLDKVHTTR-SVFDVTNVD--   234
P06608 ASPG_DICCH              180  RIGSARYITKTNASTLDTFKANEEGYLGVIIGNRIYYQNRIDKLHTTR-SVFDVRGLT--   236
Q0PC96 Q0PC96_CAMJE            159  KILSARGVVKTHSLNVDAFSSPDFGDLGYIVDGKVFFYNNVIKAHTKN-APFDVSKLT--   215
P00805 ASPG2_ECOLI             175  TVLDGRDVTKTNTTDVATFKSVNYGPLGYIHNGKIDYQRTPARKHTSD-TPFDVSKLN--   231
P10182 ASPQ_PSES7              164  EIQSGRDVSKSINIKTEAFKS-AWGPLGMVVEGKSYWFRLPAKRHTVN-SEFDIKQIS--   219
P10172 ASPQ_ACIGL              156  SIFAARDVTKGINIHTHAFVS-QWGALGTLVEGKPYWFRSSVKKHTNN-SEFNIEKIQGD   213
P50286 ASPG_WOLSU              157  EIHAAREATKLNTTAVNAFASPNTGKIGTVYYGKVEYFTQSVRPHTLA-SEFDISKIE--   213
                                      :  .*   *       :* :   * :* .   :  :     :        :

B2022031892C7BAECDB1C..         217  ALPQVDILYAYQEL-SVGMFKAAVDLGARGIVLAGLGAGFWTSKGTEEIRRIVRETKIPV   275
I1SBD9 I1SBD9_PECAS            235  KLPAVDIIYGYQDD-PEYMYDASIKHGVKGIVYAGMGAGSVSKRGDAGIRKAESK-GIVV   292
P06608 ASPG_DICCH              237  SLPKVDILYGYQDD-PEYLYDAAIQHGVKGIVYAGMGAGSVSVRGIAGMRKAMEK-GVVV   294
Q0PC96 Q0PC96_CAMJE            216  SLPKVDILYSYSNDGSGVAAKALFEHGTKGIVVAGSGAGSIHKNQKDVLKELLKK-GLKV   274
P00805 ASPG2_ECOLI             232  ELPKVGIVYNYANA-SDLPAKALVDAGYDGIVSAGVGNGNLYKSVFDTLATAAKT-GTAV   289
P10182 ASPQ_PSES7              220  SLPQVDIAYSYGNV-TDTAYKALAQNGAKALIHAGTGNGSVSSRLTPALQTLRKT-GTQI   277
P10172 ASPQ_ACIGL              214  ALPGVQIVYGSDNM-MPDAYQAFAKAGVKAIIHAGTGNGSMANYLVPEVRKLHDEQGLQI   272
P50286 ASPG_WOLSU              214  ELPRVDILYAHPDD-TDVLVNAALQAGAKGIIHAGMGNGNPFPLTQNALEKAAKS-GVVV   271
                                      ** * * *   :       .*  . *  .:: ** * *         :          :

B2022031892C7BAECDB1C..         276  IVSRRP-EGGFVGPC-------QAGIGAGFLNPQKARIQLQLALETKMDNDAIRALFEHA   327
I1SBD9 I1SBD9_PECAS            293  VRSSRT-GSGIVPPDAG-----QPGLVADSLSPAKSRILLMLALTKTTNPAVIQDYFHAY   346
P06608 ASPG_DICCH              295  IRSTRT-GNGIVPPDEE-----LPGLVSDSLNPAHARILLMLALTRTSDPKVIQEYFHTY   348
Q0PC96 Q0PC96_CAMJE            275  VVSSRV-VAGCVAV--SDSDEKLGFISAEDLNPQKARVLLMLALTKTSDPKKIQEYFLKY   331
P00805 ASPG2_ECOLI             290  VRSSRV-PTGATTQDAEVDDAKYGFVASGTLNPQKARVLLQLALTQTKDPQQIQQIFNQY   348
P10182 ASPQ_PSES7              278  IRSSHVNQGGFVLRNAEQPDDKNDWVVAHDLNPEKARILVELAMVKTQDSKELQRIFWEY   337
P10172 ASPQ_ACIGL              273  VRSSRV-AQGFVLRNAEQPDDKYGWIAAHDLNPQKARLLMALALTKTNDAKEIQNMFWNY   331
P50286 ASPG_WOLSU              272  ARSSRV-GSGSTTQEAEVDDKKLGFVATESLNPQKARVLLMLALTKTSDREAIQKIFSTY   330
                                       * :    * .             : :  *.* ::*: : **:  . :   ::  *

B2022031892C7BAECDB1C..         328  GVH                                                             330
I1SBD9 I1SBD9_PECAS            347  ---                                                             346
P06608 ASPG_DICCH              349  ---                                                             348
Q0PC96 Q0PC96_CAMJE            332  ---                                                             331
P00805 ASPG2_ECOLI             349  ---                                                             348
P10182 ASPQ_PSES7              338  ---                                                             337
P10172 ASPQ_ACIGL              332  ---                                                             331
P50286 ASPG_WOLSU              331  ---                                                             330
```

Supplementary Figure S2.

Top 5 final models from C-I-TASSER

Click to view	Rank[a]	Download	C-score[b]	Estimated TM-score[c]	Estimated RMSD[c]
	1	model1.pdb.gz	1.59	0.94±0.05	3.2±2.3Å
	2	model2.pdb.gz	-3.52		
	3	model3.pdb.gz	-4.10		
	4	model4.pdb.gz	-4.99		
	5	model5.pdb.gz	-4.99		

(a) C-I-TASSER simulations generate a large ensemble of structural conformations, i.e. decoys. These decoys are clustered by SPICKER based on pairwise structure similarity to report up to five final models from the five largest clusters. Models are ranked in descending order of cluster size. If the simulations converge well, it is possible to have less than 5 models generated, which is usually an indication of good model quality.

(b) The model confidence is quantitatified by C-score, calculated based on significance of threading template alignments, convergence of C-I-TASSER simulations and contact map satisfaction rate. C-score is typically in the range of [-5, 2], with higher C-score signifies higher model confidence.

(c) Model TM-score and RMSD are estimated based on C-score and protein length for the first model.

Supplementary Figure S3.

Predicted contact map used in C-I-TASSER simulation

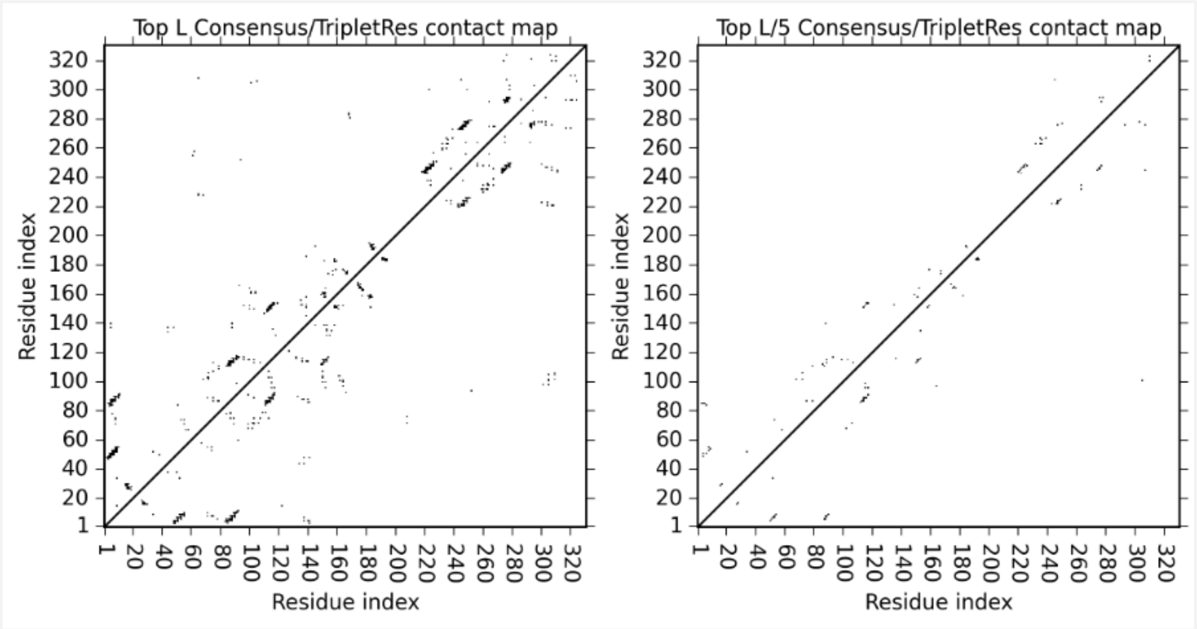

C-I-TASSER simulation is guided by consensus contact map (upper left triangle) derived based on confidence scores of TripletRes (lower right triangle), ResTriplet, ResPRE, and NeBcon. In the contact map, the axes mark the residue indexes along the sequence, while each dot represents a residue pair with predicted contact. No dot is close to the diagonal, because C-I-TASSER does not consider contacts for residue pairs separated by <6 residues.

Supplementary Figure S4.

Rank	PDB hit	ID1	ID2	Cov	Norm. Zscore	Download alignment	20 40 60 80 100 120 140 160 180 200 220<br>240 260 280 300 320
							SS: CCCSSSSSSCCCCCCSCSCCCCCCCCCCCCCCCHHHHHHHCHHHCCCCSSSSSSSSCCCCCCCCHHHHHHHHHHHHHHHHCCCCCSSSSSCCCCHHHHHHHHHHHHHHCCCCCSSSSCCCCCCCCCCCHHHHHHHHHHHHHHCCCCCCCSSSSSSCCSSSSCCCSSSCCCCCCCCCCCCCCCCSSSSSCCSSSSSCCCCCCCCCCCCCCCCCCCCCCCCCCCSSSSSCCCCCHHHHHH<br>HHHCCCCSSSSSSSCCCCCCHHHHHHHHHHHHHCCCSSSSSSCCCCCSSCCCCCSSSCCCCCHHHHHHHHHHHHHCCCCHHHHHHHHHHHCCC<br>Seq: NLPNITIYATGGTIAGSASSADQTTGYQSAALSVQSLIDAVPQLCNVANVRGVQFANTDSIDMSSDMLQALVEQIQADLDNPSTQGAVVTHGTDTLDESAFFLDLTIQSDKPVVVTGSMRPATAISADGPMNLLSSVTLAANENARGRGVMIAINDRIGSARFTTKVNANHLDAFQAPDSGLLGTFVNIQPVFFYPPSRPLGHHHFKLPPRPPSAAALPQVDILYAYQELSVGMFKA<br>AVDLGARGIVLAGLGAGFWTSKGTEEIRRIVRETKIPVIVSRRPEGGFVGPCQAGIGAGFLNPQKARIQLQLALETKMDNDAIRALFEHAGVH
1	7p9cA	0.40	0.38	11.34	**3.94**	SPARKS-K	-LPNITILATGGTIAGGGDSATKS------NVGVENLVNAVPQLKDIANVKGEQVVNIGSQDMNDNVWLTLAKKINTDCDKT--DGFVITHGTDTMEETAYFLDLTVKCDKPVVMVGAMRPSTSMSADGPFNLYNAVVTAADKASANRGVLVVMNDTVLDGRDVTKTNTTDVATFKSVNYGPLGYIHNGKIDYQ---<br>RTPARKHTSDTPFDVSKLNELPKVGIVYNYANASDLPAKALVDAGYDGIVSAGVGNGNLYKSVFDTLATAAKT-GTAVVRSSRVPTGATTQDAGFVASGTLNPQKARVLLQLALTQTKDPQQIQQIFNQY---
2	1hfjA	0.46	0.45	13.17	**4.00**	FFAS-3D	KLPNIVILATGGTIAGSAATGTQTTGYKAGALGVDTLINAVPEVKKLANVKGEQFSNMASENMTGDVVLKLSQRVNELLARDDVDGVVITHGTDTVEESAYFLHLTVKSDKPVVFVAAMRPATAISADGPMNLLEAVRVAGDKQSRGRGVMVVINDRIGSARYITKTNASTLDTFRANEEGYLGVIIGNRIYYQ---<br>NRIDKLHTTRSVFDVRGLTSLPKVDILYGYQDDPEYLYDAAIQHGVKGIVYAGMGAGSVSVRGIAGMRKALEK-GVVVMRSTRTGNGIVPPDLPGLVSDSLNPAHARILLMLALTRTSDPKVIQEYFHTY---
3	2jk0A	0.47	0.46	13.41	**12.45**	HHpred	NLPNIVILATGGTIAGSAAANTQTTGYKAGALGVETLIQAVPELKTLANIKGEQVASIGSENMTSDVLLTLSKRVNELLARSDVDGVVITHGTDTLDESPYFLNLTVKSDKPVVFVAAMRPATAISADGPMNLYGAVKVAADKNSRGRGVLVVLNDRIGSARFISKTNASTLDTFKAPEEGYLGVIIGDKIYYQTRLDKVTTRSVFDVT----<br>NVDKLPAVDIIYGYQDDPEYMYDASIKHGVKGIVYAGMGAGSVSKRGDAGIRKAESK-GIVVVRSSRTGSGIVPPGQPGLVADSLSPAKSRILLMLALTKTTNPAVIQDYFHAY---
4	2jk0A	0.47	0.46	13.42	**2.97**	MUSTER	NLPNIVILATGGTIAGSAAANTQTTGYKAGALGVETLIQAVPELKTLANIKGEQVASIGSENMTSDVLLTLSKRVNELLARSDVDGVVITHGTDTLDESPYFLNLTVKSDKPVVFVAAMRPATAISADGPMNLYGAVKVAADKNSRGRGVLVVLNDRIGSARFISKTNASTLDTFKAPEEGYLGVIIGDKIYYQTRLDKV---<br>HTTRSVFDVTNVDKLPAVDIIYGYQDDPEYMYDASIKHGVKGIVYAGMGAGSVSKRGDAGIRKAESK-GIVVVRSSRTGSGIVPPDQPGLVADSLSPAKSRILLMLALTKTTNPAVIQDYFHAY---
5	5i4bA	0.46	0.45	13.09	**4.04**	CNFpred	KLPNIVILATGGTIAGSAATGTQTTGYKAGALGVDTLINAVPEVKKLANVKGEQFSNMASQNMTGDVVLKLSQRVNELLARDDVDGVVITHGTDTVEESAYFLHLTVKSDKPVVFVAAMRPATAISADGPMNLLEAVRVAGDKQSRGRGVMVVLNDRIGSARYITKTNASTLDTFKANEEGYLGVIIGNRIYYQNRI---<br>DKLHTTRSVFDVRGLTSLPKVDILYGYQDDPEYLYDAAIQHGVKGIVYAGMGAGNVSVRGIAGMRKAME-KGVVVIRSTRTGNGIVPPDLPGLVSDSLNPAHARILLMLALTRTSDPKVIQEYFHTY---
6	2jk0A	0.47	0.46	13.41	**1.54**	HHsearch-2	NLPNIVILATGGTIAGSAAANTQTTGYKAGALGVETLIQAVPELKTLANIKGEQVASIGSENMTSDVLLTLSKRVNELLARSDVDGVVITHGTDTLDESPYFLNLTVKSDKPVVFVAAMRPATAISADGPMNLYGAVKVAADKNSRGRGVLVVLNDRIGSARFISKTNASTLDTFKAPEEGYLGVIIGDKIYYQTRLDKVHTRSVFDVT----<br>NVDKLPAVDIIYGYQDDPEYMYDASIKHGVKGIVYAGMGAGSVSKRGDAGIRKAE-SKGIVVVRSSRTGSGIVPPDQPGLVADSLSPAKSRILLMLALTKTTNPAVIQDYFHAY---
7	2jk0A	0.47	0.46	13.42	**3.70**	Neff-PPAS	NLPNIVILATGGTIAGSAAANTQTTGYKAGALGVETLIQAVPELKTLANIKGEQVASIGSENMTSDVLLTLSKRVNELLARSDVDGVVITHGTDTLDESPYFLNLTVKSDKPVVFVAAMRPATAISADGPMNLYGAVKVAADKNSRGRGVLVVLNDRIGSARFISKTNASTLDTFKAPEEGYLGVIIGDKIYYQTRLDKV---<br>HTTRSVFDVTNVDKLPAVDIIYGYQDDPEYMYDASIKHGVKGIVYAGMGAGSVSKRGDAGIRKAESKG-IVVVRSSRTGSGIVPPDQPGLVADSLSPAKSRILLMLALTKTTNPAVIQDYFHAY---
8	2jk0A	0.46	0.45	13.17	**2.46**	HHsearch	NLPNIVILATGGTIAGSAAANTQTTGYKAGALGVETLIQAVPELKTLANIKGEQVASIGSENMTSDVLLTLSKRVNELLARSDVDGVVITHGTDTLDESPYFLNLTVKSDKPVVFVAAMRPATAISADGPMNLYGAVKVAADKNSRGRGVLVVLNDRIGSARFISKTNASTLDTFKAPEEGYLGVIIGDKIYYQTRLDKVHTT---<br>RSVFDVTNVDKLPAVDIIYGYQDDPEYMYDASIKHGVKGIVYAGMGAGSVSKRGDAGIRKAE-SKGIVVVRSSRTGSGIVPPQPGL-VADSLSPAKSRILLMLALTKTTNPAVIQDYFHAY---
9	7p9cA	0.41	0.39	11.42	**3.00**	PROSPECTOR2	-LPNITILATGGTIAGGGD------SATKSNVGVENLVNAVPQLKDIANVKGEQVVNIGSQDMNDNVWLTLAKKINTDCDK--TDGFVITHGTDTMEETAYFLDLTVKCDKPVVMVGAMRPSTSMSADGPFNLYNAVVTAADKASANRGVLVVMNDTVLDGRDVTKTNTTDVATFKSVNYGPLGYIHNGKIDYQRTPARK---<br>HTSDTPFDVSKLNELPKVGIVYNYANASDLPAKALVDAGYDGIVSAGVGNGNLYKSVFDTLATAA-KTGTAVVRSSRVPTGATTQDAEVVASGTLNPQKARVLLQLALTQTKDPQQIQQIFNQ---Y
10	2d6fA	0.24	0.23	7.09	**4.44**	SAM	-LPDVSIISTGGTVASIIDYRT---GAVHPAFTADDLLRANPELLDIANIRGRAVFNILSENMKPEYWVETARAVYGEIKD-GADGVVAHGTDTMHYTSAALSFMLRTPVPVVFTGAQRSSDRPSSDASLNIQCSVRAATSEI---AEVTVCMHAHLHRGVKVRKMHTSRRDTFRSMNALPLAEVTPDGIKILEENYRKRG-----<br>SDELELSDRVEERVAFIKSYPGISPDIIKWHLDEGYRGIVIEGTGLGHCPDTLIPVI-GEAHDMGVPVAMTSQCLNGRVLLQAGVIPCDDMLPEVAYVKMCWVLGQTDDPEMAREMMRENIAG

(a) *ID1* is the number of template residues identical to query divided by number of aligned residues.

(b) *ID2* is the number of template residues identical to query divided by query sequence length.

(c) *Cov* is equal the number of aligned template residues divided by query sequence length.

(d) *Norm. Zscore* is the normalized Z-score of the threading alignments. A Normalized Z-score >1 means a good alignment and is highlighted in **bold**.

(e) *Download alignment* lists the threading program used to identify the template, and provide the 3D structure of aligned regions of threading templates (threading[1-10].pdb.gz).

(f) Template residues identical to query sequence are highlighted in color.
